# Overlapping Stromal Alterations in Myeloid and Lymphoid Neoplasms

**DOI:** 10.3390/cancers16112071

**Published:** 2024-05-30

**Authors:** Lucienne Bogun, Annemarie Koch, Bo Scherer, Ulrich Germing, Roland Fenk, Uwe Maus, Felix Bormann, Karl Köhrer, Patrick Petzsch, Thorsten Wachtmeister, Guido Kobbe, Sascha Dietrich, Rainer Haas, Thomas Schroeder, Stefanie Geyh, Paul Jäger

**Affiliations:** 1 Department of Hematology, Oncology and Clinical Immunology, Medical Faculty, University of Duesseldorf, 40225 Duesseldorf, Germany; lucienne.bogun@uni-duesseldorf.de (L.B.); annemarie.koch@med.uni-duesseldorf.de (A.K.); bo.scherer@med.uni-duesseldorf.de (B.S.); germing@med.uni-duesseldorf.de (U.G.); fenk@med.uni-duesseldorf.de (R.F.); kobbe@med.uni-duesseldorf.de (G.K.); sascha.dietrich@med.uni-duesseldorf.de (S.D.); haas@med.uni-duesseldorf.de (R.H.); thomas.schroeder@uk-essen.de (T.S.); 2 Department of Orthopedic Surgery and Traumatology, Medical Faculty, University of Duesseldorf, 40225 Duesseldorf, Germany; uwe.maus@med.uni-duesseldorf.de; 3 Bioinformatics.Expert UG, 12305 Berlin, Germany; f.bormann@bioinformatics.expert; 4 Biological and Medical Research Center (BMFZ), Medical Faculty, Heinrich-Heine-University, Universitätsstraße 1, 40225 Düsseldorf, Germany; koehrer@uni-duesseldorf.de (K.K.); patrick.petzsch@hhu.de (P.P.); thorsten.wachtmeister@uni-duesseldorf.de (T.W.)

**Keywords:** myeloid neoplasms, lymphoid neoplasms, MPN, MDS, AML, ALL, NHL, MSC, bone marrow microenvironment, osteogenesis, hematopoietic insufficiency, RNA sequencing

## Abstract

**Simple Summary:**

**Simple Summary:** Myeloid and lymphoid malignant cells can become the dominant population in the bone marrow and thus inhibit healthy hematopoiesis. Patients suffer enormously as a result. In addition to the directly mediated inhibition of healthy hematopoietic stem and progenitor cells, indirect mechanisms via so-called mesenchymal stromal cells can also play a role. We are focusing our research on the latter and would like to identify functional and molecular overlapping mechanisms within the various myeloid and lymphoid neoplasms. In the future, it may be possible to block these mechanisms and thus prevent disease progression or improve healthy hematopoiesis.

**Abstract:**

Myeloid and lymphoid neoplasms share the characteristics of potential bone marrow infiltration as a primary or secondary effect, which readily leads to hematopoietic insufficiency. The mechanisms by which clonal malignant cells inhibit normal hematopoietic stem and progenitor cells (HSPCs) in the bone marrow (BM) have not been unraveled so far. Given the pivotal role of mesenchymal stromal cells (MSCs) in the regulation of hematopoiesis in the BM niche it is assumed that MSCs also play a relevant role in the pathogenesis of hematological neoplasms. We aimed to identify overlapping mechanisms in MSCs derived from myeloid and lymphoid neoplasms contributing to disease progression and suppression of HSPCs to develop interventions that target these mechanisms. MSCs derived from healthy donors (*n* = 44) and patients diagnosed with myeloproliferative neoplasia (*n* = 11), myelodysplastic syndromes (*n* = 16), or acute myeloid leukemia (*n* = 25) and B-Non-Hodgkin lymphoma (*n* = 9) with BM infiltration and acute lymphoblastic leukemia (*n* = 9) were analyzed for their functionality and by RNA sequencing. A reduced growth and differentiation capacity of MSCs was found in all entities. RNA sequencing distinguished both groups but clearly showed overlapping differentially expressed genes, including major players in the BMP/TGF and WNT-signaling pathway which are crucial for growth, osteogenesis, and hematopoiesis. Functional alterations in healthy MSCs were inducible by exposure to supernatants from malignant cells, implicating the involvement of these factors in disease progression. Overall, we were able to identify overlapping factors that pose potential future therapeutic targets.

## 1. Introduction

Myeloid neoplasms are mainly represented by myelodysplastic syndromes (MDSs), myeloproliferative neoplasia (MPN), or acute myeloid leukemia (AML), while lymphoid neoplasms comprise aggressive and indolent non-Hodgkin lymphoma (NHL) as well as acute lymphoblastic leukemia (ALL) [1,2]. Independent of the underlying specific genetic aberrations, these hematological neoplasms originate from hematopoietic cells at various differentiation stages, such as hematopoietic stem and progenitor cells (HSPCs) or line-determined progenitor cells. Irrespective of the molecular aberrations, in general, they confer growth and survival advantages to those cells over normal HSPCs [3,4,5].

A common feature of these neoplasms is the infiltration of the bone marrow (BM) by malignant cells, either primarily or secondarily, with a functional takeover resulting in hematopoietic insufficiency of various degrees. Consequently, patients experience cytopenia-related symptoms, including anemia, infections, and bleeding, which represent the major causes of morbidity and mortality [6,7].

The detailed underlying mechanisms by which clonal malignant cells functionally inhibit normal HSPCs in the BM are only partially understood. To some extent, the suppression of normal hematopoiesis certainly results from direct interactions between clonal malignant cells and healthy HSPCs [8]. The BM microenvironment, particularly of mesenchymal stromal cells (MSCs), plays a pivotal role in the regulation of normal hematopoiesis and their immunomodulatory potential and contributes to extracellular matrix formation. Recent work from our group, as well as other research groups, has shed some light on the pathophysiological role of the BM microenvironment in the progression and myelosuppression of hematological neoplasms [9,10,11,12,13,14].

For instance, MSCs derived from patients with AML and MDS show structural, functional, and epigenetic alterations, along with specific gene expression signatures resulting in inadequate stromal support for normal hematopoiesis [10,14,15,16,17,18].

Similar results have been reported in lymphoid malignancies. Indeed, MSCs derived from patients with multiple myeloma exhibit structural and functional abnormalities comparable to those observed in MDS and AML [9] and significantly contribute to hematopoietic insufficiency in these patients. Co-culture experiments of patient-derived ALL blasts with healthy MSCs resulted in an inhibition of osteogenic differentiation and a reduced number of osteoblastic cells [19].

In the light of potential overlaps in the phenotype, functionality, and molecular mechanisms of MSCs between myeloid and lymphoid neoplasms, we performed a comprehensive analysis including functional assessments and RNA sequencing (RNAseq) of MSCs from patients with both myeloid and lymphoid neoplasms. The identification of common pathological pathways could help to design a targeted therapy in the future to restore adequate stromal support of normal hematopoiesis.

## 2. Materials and Methods

### 2.1. Patients, Healthy Controls, and Cell Preparation

Bone marrow (BM) samples were obtained from 70 patients covering AML (*n* = 25), MDS (*n* = 16), MPN (*n* = 11), ALL (*n* = 9), and NHL (≥10% BM infiltration) (*n* = 9) who received their initial diagnosis between 2019 and 2023. A total of 44 age- and sex-matched healthy donors served as a control group (HC, median age: 67 years, range: 33–86 years). Detailed patient characteristics are given in Table 1.

MSCs were derived from the mononuclear cell (MNC) fraction of these specimens and cultured as previously described [10]. All experiments were carried out using MSCs derived from passages 3–4. Furthermore, in 19 cases of these patients, MSCs as well as MNCs for generations of conditioned media were available.

### 2.2. Cell Culture Conditions and Reagents

MSCs were cultured as previously described [20]. MNC fractions were cultured in RPMI + 20% FBS (Sigma Aldrich, St. Louis, MO, USA) and supplemented with IL-3, IL-6, SCF (all 10 ng/mL), and FLT-3Ligand (20 ng/mL), all from Peprotech, Hamburg, Germany, as previously described [8].

### 2.3. Growth Properties and Cellular Senescence

The colony-forming unit fibroblast (CFU-F) activity was determined in primary culture under a light microscope. Cumulative population doublings (CPDs) were calculated after each passage. ß-galactosidase activity, as an indicator for senescent cells was measured using the Cellular Senescence Detection Kit (Biolabs, San Diego, CA, USA) in accordance with the manufacturers’ instructions. Under a light microscope, visualization of senescent cells, as reflected by their blue staining through ß-galactosidase activity, was evaluated as previously described [10]. 

### 2.4. Conditioned Media

To generate conditioned media (CM), 2.7 × 10^4^/cm^2^ cells of the cell lines NALM-6, MAVER-1, MEC-1, HL-60, and K422 (all purchased from DSMZ, Braunschweig, Germany) were cultivated in T75 cm^2^ culture flasks for 3 days following the manufacturers’ instructions. For patient-derived CM, 2 × 10^5^/cm^2^ BM-derived MNCs from patients with myeloid neoplasms (MPN, MDS, and AML) and lymphoid neoplasms (ALL and NHL) and MNCs (*n* = 16) from healthy controls (HC) were cultivated in RPMI + 20% FBS medium supplemented with IL-3, IL-6, SCF (10 ng/mL), and FLT3-Ligand (20 ng/mL, all from Peprotech, Hamburg, Germany). Cells were cultured under humidified conditions at 37 °C and 5% CO_2_. After incubation time, cell-free CM was harvested by filtration (Minisart^®^ 0.45 μm, Sartorius AG, Göttingen, Germany) and stored at −80 °C for further experiments.

### 2.5. Differentiation Properties

Differentiation assays from native MSCs into adipocytes, chondroblasts, and osteoblasts were performed on Passage 3. For adipogenic differentiation, DMEM high glucose was supplemented with Insulin (0.1 mg/mL), Indomethacin (0.2 mM), Isobutylmethylxanthane (1 mM), and Dexamethasone (10^−6^ M) (all Sigma Aldrich), and differentiated for 21 days. Visualization of lipid vacuoles was performed using Oil Red O staining. Chondrogenic differentiation was induced with DMEM high glucose supplemented with TGF-ß3 (10 ng/mL, PeproTech Inc., Rocky Hill, CT, USA), L-Proline (40 µg/mL), ascorbate-2-phosphate (50 µg/mL), ITS+1 (1%) and Dexamethasone (10^−7^ M) (All from Sigma Aldrich). After 21 days, chondrogenic pellets were cut on a cryostate, and proteoglycane was stained with Safranin O. Alizarin Red staining was used to visualize osteogenic differentiation after 14 days of osteogenic induction in a medium consisting of DMEM low glucose ascorbic acid (50 µg/mL), ß-glycerol-phosphate (10 mM) and dexamethasone (10^−7^ M). Differentiation assays were performed as previously published [10,20]. Images were digitalized using SPOT Software version 4.7 (Diagnostic Instruments Inc., Sterling Heights, MI, USA) as previously described [10] and visualized using an Axiovert 25 microscope (Zeiss, Jena, Germany). We used a 5 objective-Zeiss CP-Achromat 5 Ph0 for native and osteogenic differentiated MSCs, a 10 objective-Zeiss CP-Achromat 10 Ph1 for adipogenic differentiated MSCs, and a 2.5 objective-Zeiss CP Achromat for chondrogenic differentiation. 

### 2.6. Hematopoietic Support—Long-Term Culture-Initiating Cells (LTC-IC Assay)

A total of 0.8 × 10^6^–1.0 × 10^6^ MSCs were cultivated on 96-well plates (Costar, Corning, KS, USA) and irradiated with 30 Gray using Gulmay RS225 X-ray equipment. Subsequently, 6 × 10^3^ healthy CD34+ cells were plated on these MSC feeder layers and then further processed using the same conditions and reagents as in our previous work [10,15].

### 2.7. Co-Culture with Conditioned Media and Blocking of TGFB1

To investigate which properties are mostly affected on healthy MSCs due to the malignant compartment, 1.4 × 10^4^/cm^2^ healthy MSCs were cultivated with primary MDS, MPN, AML, ALL or MM-derived MNCs, or cell line-derived CM (generated as described in 2.4) from INA-6, RPMI-8226, NALM-6, MEC-1, MAVER-1, SU-DHL-6, and K-422 on a 6-well plate. After 72 h (at 37 °C, 5% CO_2_ under humidified conditions), viable cells were counted under a light microscope and were included in the following experiments.

Using SD208, an active ATP-competitive transforming growth factor-β receptor 1 (TGF-βRI) (ALK5) inhibitor (0.5 µM diluted in 10 mM DMSO, Biotechne/R&D systems, Minneapolis, Minnesota) to investigate the effects on TGFB1 signaling, healthy MSCs and MSCs derived from myeloid and lymphoid neoplasms were induced towards osteogenic differentiation and SD208 or DMSO (as control) was added to each medium change during the 14 days of osteogenic induction.

### 2.8. Quantitative Realtime-Polymerase Chain Reaction (qRT-PCR)

Total RNA was isolated using the RNeasy Micro Kit or Mini Kit (Qiagen, Hilden, Germany) with the optional DNase digestion, following the manufacturer’s instructions. Using a StepOne Plus Realtime PCR cycler and Power SYBR Green PCR Mastermix (Applied Biosystem, Life Technologies, Carlsbad, CA, USA), qRT-PCR was performed in duplicate. GAPDH was used as reference control and differences in mRNA expression level were evaluated with the ∆∆CT method. Primer sequences can be provided on request.

### 2.9. RNA-Sequencing

For transcriptome analysis, total RNA was DNase digested and quality controlled before further use by quantifying them fluorometrically (Qubit RNA HS Assay, Thermo Fisher Scientific Inc., Waltham, MA, USA) and checking their integrity by capillary gel electrophoresis on the FragmentAnalyzer system using the “Total RNA Standard Sensitivity Assay” (Agilent Technologies, Inc., Santa Clara, CA, USA). Libraries were prepared using the VAHTS universal V6 RNA-seq library prep kit (Illumina, San Diego, CA, USA). Briefly, 500 ng total RNA was used for poly(A) RNA selection, fragmentation, cDNA generation, adapter ligation, strand selection, and library amplification. Bead-purified libraries were normalized and finally sequenced on the NextSeq 2000 system (Illumina Inc. San Diego, CA, USA) with a read setup of 1 × 151 bp. The bcl2fastq tool was used to convert the bcl files to fastq files and for adapter trimming and demultiplexing as previously described [8].

### 2.10. Bioinformatical Analysis

Bioinformatical analysis A: The fastq files were analyzed with CLC Genomics Workbench (version 20.0.3, QIAGEN, Venlo, The Netherlands). The reads of the samples were both adapter and quality trimmed. The default settings were used (bases < Q13 were trimmed from the end of the reads, ambiguous nucleotides max. 2). The reads were mapped against the human reference genome (hg38, GRCH38.103) (14 April 2021). Multi-group comparisons of the individual conditions (3 replicates each) were performed and statistically analyzed. The Empirical Analysis of DGE (version 1.1, cutoff = 5) algorithm implemented in CLC was used for this purpose. A *p*-value < 0.05 was considered significant after multiple test corrections for FDR. Using the Ingenuity Pathway Analysis software (Qiagen Inc., 2020, Venlo, The Netherlands), the data were further analyzed while maintaining the standard parameters (|FC| ≥ 1.5, *p* ≤ 0.01, P [FDR] ≤ 0.05).

Bioinformatical analysis B: Raw sequencing reads were cropped at their 5′ position by 12 bases using the Trimmomatic function “headcrop”. The following analyses were initiated in the first quarter of 2023 using the online resources listed below. Alignment was then performed in a two-pass approach with STAR v2.7.2 [18] (adapted from https://docs.gdc.cancer.gov/Data/Bioinformatics_Pipelines/Expression_mRNA_Pipeline and described previously) [8]. New splice junctions were detected in the first pass and added to the reference human genome GRCh38.97 (hg38) to complement the second pass alignment. Subsequently, featureCounts (1.6.5) and DESeq2 were applied to the aligned reads to identify differentially expressed genes. An FDR q-value of below 0.05 was used as the criterion to assign significance. Additionally, StringTie (2.0.3) [19] was applied to calculate fragments per kilobase of transcript per million mapped reads (FPKM-values). Further downstream analyses contained principal component analysis using the R package FactoMineR (1.42) and heatmaps created with pheatmap (1.0.12). Gene set enrichment analysis (GSEA, Broad Institute, Boston, MA, USA) was tested with FPKM values on custom gene sets or gene sets within the Molecular Signature Database (https://www.gsea-msigdb.org/gsea/msigdb). Gene Ontology analysis was conducted using a Panther over-representation test (https://amigo.geneontology.org/amigo) using the significantly differentially expressed genes as input. Significance was addressed by a Fisher’s exact test followed by a Bonferroni correction.

### 2.11. Data Access

RNA sequencing expression data are currently being deposited in the NCBI Sequence Read Archive (SRA) with BioProject ID PRJNA1091937.

### 2.12. Statistical Analysis

GraphPad Prism 8.4.3 (GraphPad Software Inc., La Jolla, CA, USA) was used for statistical analyses. The Wilcoxon signed-rank test was used for intraindividual analysis, while a two-sided unpaired Student’s *t*-test was performed for interindividual comparison. SEM and means are provided for every experiment and a statistically significant level of *p* ≤ 0.05 was determined (* *p* ≤ 0.05; ** *p* ≤ 0.01; *** *p* ≤ 0.001; **** *p* ≤ 0.0001).

## 3. Results

### 3.1. MSCs from Myeloid and Lymphoid Neoplasms Showed Similar Alterations in Growth Capacity and Cellular Senescence

**Figure 1:**
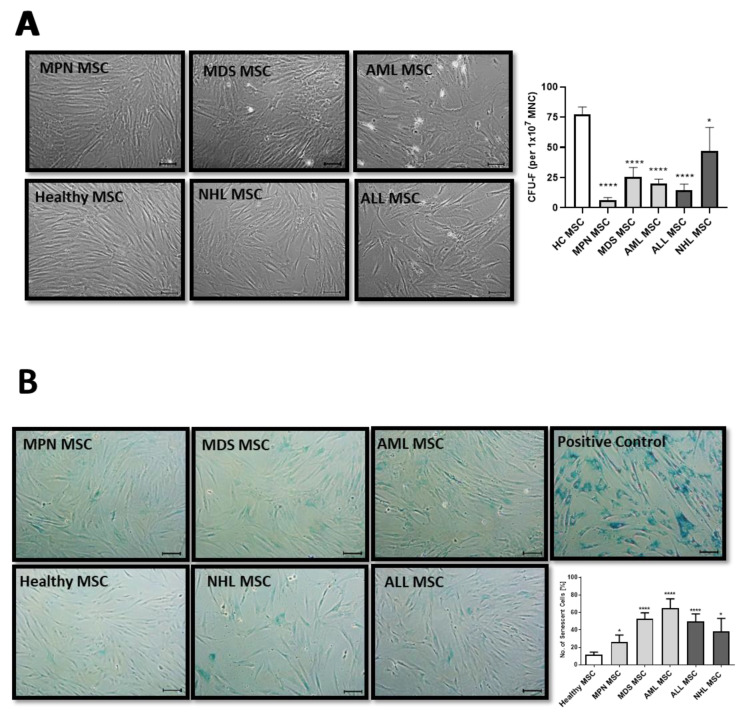
Growth capacity and cellular senescence of MSCs from myeloid and lymphoid neoplasms. (**A**) Representative micrographs of phenotype from Healthy-, MPN-, MDS-, AML-, ALL-, and NHL-derived MSCs with scale bars indicating 100 µm are shown. Bar charts of the CFU-F activity of MSCs from Healthy-, MPN-, MDS-, AML-, ALL-, and NHL-derived MSCs. (**B**) Representative micrographs of number of cellular senescence visualized in blue from native Healthy-, MPN-, MDS-, AML-, ALL-, and NHL-derived MSCs in Passage 3 after the ß-galactosidase staining. Scale bars indicating 100 µm are shown. Asterisks display *p*-values * *p* < 0.05, **** *p* < 0.0001.

MSCs are usually characterized in vitro by a spindle-type morphology, their colony-forming unit fibroblast (CFU-F) activity, and plastic adherence, which results in the formation of a parallel growing feeder layer reaching 80% confluence after a few days in culture. Our group and others have previously reported deficits in morphology, which were accompanied by a diminished growth capacity as well as a higher percentage of cellular senescence, especially for MDS and AML-derived MSCs [10,15]. In contrast, MSCs derived from MPN, ALL, and NHL displayed a less atypical phenotype when compared to the disturbed morphology observed in MDS and AML-derived MSCs from new samples obtained for this comprehensive study (Figure 1A). Moreover, MDS and AML-derived MSCs were more prone to cellular senescence, as reflected by active ß-galactosidase in contrast to the relatively lower incidence observed in MPN, ALL, or NHL-derived MSCs (Figure 1B).

### 3.2. MSCs from Myeloid and Lymphoid Neoplasms Showed Similar Reduced Chondrogenic-Osteogenic Differentiation Capacity

**Figure 2:**
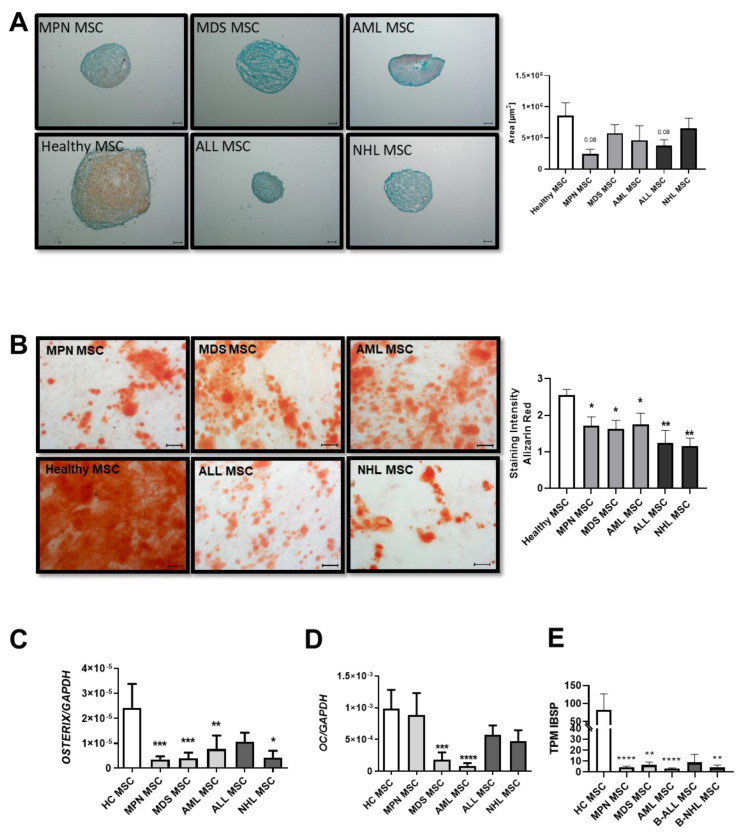
Chondrogenic and osteogenic differentiation capacity of MSCs derived from patients with myeloid and lymphoid neoplasms. (**A**) Representative micrographs of proteoglycan after Safranin O staining of chondrogenic-induced MSCs after 21 days. Scale bars indicating 100 µm are shown. Right side: area size in µm of chondrogenic pellets after 21 days of induction was measured and presented as bar charts of the respective MSC group in comparison to healthy MSCs (white bar). (**B**) Osteogenic differentiation was induced for 14 days and stained with Alizarin Red. Representative micrographs of the osteogenic potential of MSCs. Scale bars indicating 100 µm are shown. Right side: Bar charts represent staining intensity of observed osteogenic differentiation. Osteogenic differentiation capacity was graded according to the microscope as previously described [10]. Bar charts of the mRNA expression level of osteogenic factors *OSTERIX* (**C**), *OSTEOCALCIN* (**D**), and (**E**) TPM values from our sequencing data of Integrin Binding Sialoprotein (IBSP). For all other experiments, results are expressed as Mean ± SEM. Asterisks display *p*-values * *p* < 0.05, ** *p* < 0.01, *** *p* < 0.001, **** *p* < 0.0001.

Patient-derived MSCs from all groups showed a reduced chondrogenic differentiation capacity, as evidenced by the pellet size and safranin O staining. In detail, MDS-derived MSCs showed the most severely reduced chondrogenic differentiation capacity, visualized by Safranin O and a very rough and cracking surface. MPN and AML-derived MSCs displayed a rather normal smooth chondrogenic surface structure, albeit with an uneven distribution of proteoglycan (Figure 2A). Conversely, ALL-derived MSCs exhibit the highest amount of proteoglycan despite their reduced pellet size, while NHL-derived MSCs showed a similar reduced chondrogenic differentiation capacity to that of MDS-derived MSCs (Figure 2A).

MSCs derived from MDS and AML showed a 1.4-fold reduced osteogenic differentiation capacity (MDS: 1.8; AML: 1.75 vs. HC: 2.6, *p* value: ≤0.05), as already published. However, a significantly greater reduction of 2-fold with regard to osteogenic differentiation capacity was seen in MSCs derived from MPN patients (MPN: 1.3 vs. HC: 2.6 *p*-value ≤ 0.01). Even more pronounced, ALL-derived MSCs showed a 2.6-fold reduced osteogenic capacity in comparison to MSCs derived from healthy individuals, while NHL-derived MSCs showed the greatest osteogenic impairment with a 3.2-fold diminished differentiation capacity (ALL: 1 and NHL: 0.8 vs. HC: 2.6, *p*-values ≤ 0.01) (Figure 2B). Molecular analysis revealed a correlation with decreased expression levels of osteogenic factors such as OSTERIX or OSTEOCALCIN in qRT-PCR analysis. A significantly decreased expression of OSTERIX was measured in all patient-derived MSCs (Figure 2C), while OSTEOCALCIN was strongly reduced in MPN, ALL, and NHL-derived MSCs, and significantly reduced in MDS and AML-derived MSCs (Figure 2D). Furthermore, TPM values of IBSP from our sequencing data were strongly decreased in derived MSCs (Figure 2E).

Using Oil Red O staining, no significant differences in adipogenic differentiation capacity could be observed in adipogenic-induced MSCs after 21 days of culture. 

### 3.3. Insufficient Hematopoietic Support in Myeloid and Lymphoid Neoplasms

**Figure 3:**
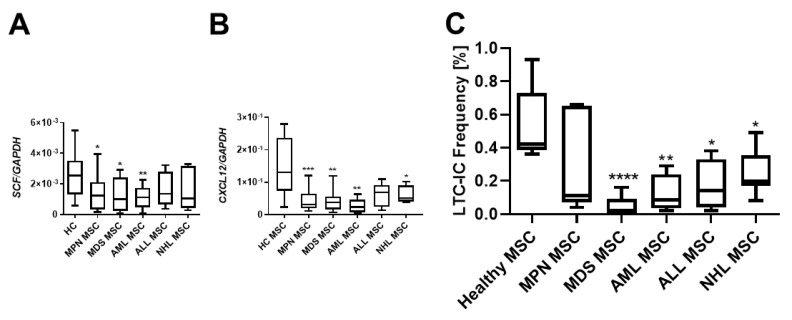
Hematopoietic supporting capacity of MSCs from myeloid and lymphoid neoplasms. Box plots of mRNA expression of hematopoietic factors SCF (**A**) and SDF-1 (*CXCL12*) (**B**) in healthy-, MPN-, MDS-, AML-, ALL-, and NHL-derived MSCs. mRNA expression was measured by qRT-PCR in healthy MSCs (*n* = 22), MPN MSCs (*n* = 14), MDS MSCs (*n* = 10), AML MSCs (*n* = 10), ALL MSCs (*n* = 7), and NHL MSCs (*n* = 4). (**C**) Box plots of long-term initiating cell (LTC-IC) assay frequency. Hematopoietic supporting capacity of MSCs derived from healthy and hematological neoplasms. Asterisks display *p*-values * *p* < 0.05, ** *p* < 0.01, *** *p* < 0.001, **** *p* < 0.0001.

Crucial interactions governing the homing, proliferation, or differentiation of HSPCs with MSCs are well established. Stem Cell Factor (*SCF*), also known as *KITLG*, plays a pivotal role in the differentiation, proliferation, and survival of hematopoietic stem cells. Notably, *SCF* was found to be strongly downregulated in MSCs over all hematological neoplasms, and MSCs derived from myeloid neoplasms exhibited the most pronounced reduced mRNA expression of SCF (Figure 3A). The stromal cell-derived factor-1 (*SDF-1*, also known as *CXCL12*) is another key player in hematopoiesis, directing migrating HSPCs or activating lymphocytes. *SDF-1* mRNA expression was striking diminished in all MSCs from patients with hematological neoplasms, encompassing both myeloid and lymphoid (Figure 3B). In terms of functionality assessed in a long-term culture initiating cell (LTC-IC) assay, decreased expression of SCF and SDF-1 was associated with decreased hematopoietic supporting capacity. In detail, MDS and AML MSCs exhibited a 9.2-fold and a 7.6-fold reduced hematopoietic supporting capacity, while MPN MSCs showed a 1.2-fold, ALL-derived MSCs a 2.1-fold, and NHL-derived MSCs a 2.4-fold reduced hematopoietic supporting capacity (Figure 3C, LTC-IC frequency in %, HC: 0.55%; MPN: 0.31%, MDS: 0.05% *p*-value: ≤0.05; AML: 0.12% *p*-value: ≤0.05; ALL: 0.238; NHL: 0.19% *p*-values: ≤0.05).

### 3.4. Patient-Derived and Cell Line-Derived Conditioned Media-Induced Deficits in MSCs

Previous data from our group and others have indicated that conditioned media from AML cell lines could induce various alterations in MSCs related to growth and differentiation [4,20]. Building upon the hypothesis that soluble factors contribute to stromal alterations, we exposed healthy MSCs to conditioned Media (CM) derived from MNCs of healthy controls and patients with MDS, AML, ALL, and NHL. Additionally, we use CM from commercial cell lines covering AML HL-60, ALL NALM-6, and NHL cell lines MEC-1, MAVER-1, and K422.

**Figure 4:**
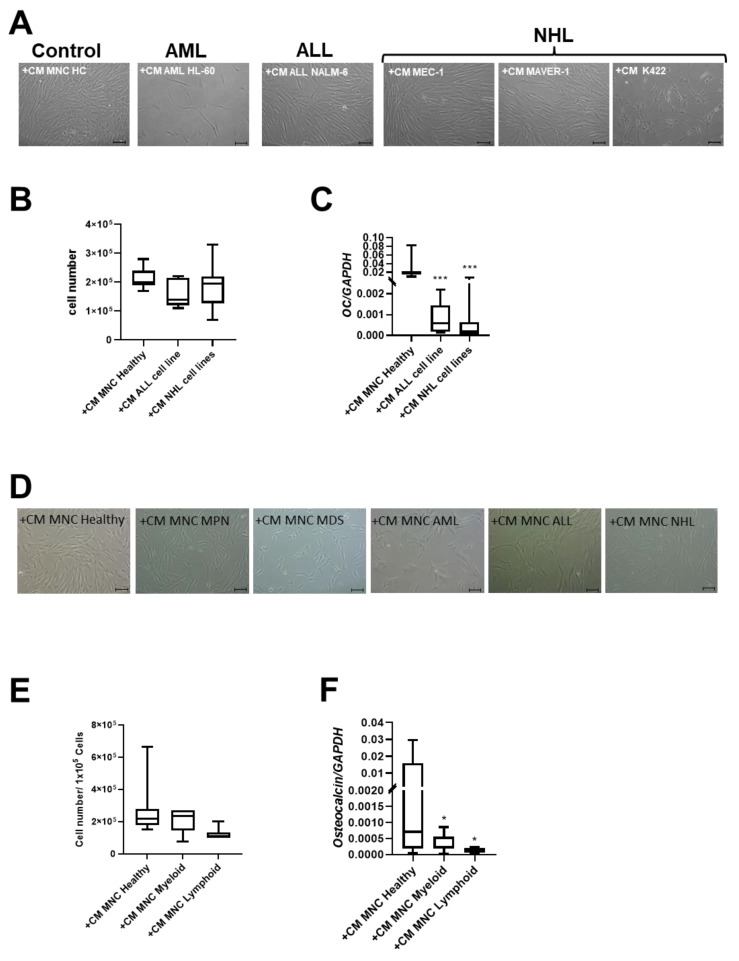
Exposure of healthy MSCs to conditioned media (CM) derived from cell lines and BM-MNCs from patients with myeloid and lymphoid neoplasms. (**A**) Representative micrographs of phenotype of healthy MSCs after co-culture with CM from cell line HL-60 (AML), cell line NALM-6 (ALL), MEC-1, MAVER-1, and K422 (all three NHL). Scale bars indicating 100 µm are shown. (**B**) Box plots present cell numbers of healthy MSCs after 3 days of co-culture with CM derived from cell lines. (**C**) Box plots demonstrate mRNA expression of osteogenic factor *OSTEOCALCIN* measured by qRT-PCR. (**D**) Representative micrographs of phenotype of healthy MSCs after co-culture with patient-derived CM from BM-derived MNCs for 3 days. Scale bars indicating 100 µm are shown. Calculated cell numbers of manipulated healthy MSCs after 3 days of co-culture are given in box plots. (**E**) Box plots present cell numbers of healthy MSCs after 3 days of co-culture with CM derived from patients’ BM MNCs. (**F**) mRNA expression of *OSTEOCALCIN* was measured in healthy MSCs after co-culture with respective patients BM-MNC CM by qRT-PCR. Asterisks display *p*-values * *p* < 0.05, *** *p* < 0.001.

After 3 days of cultivation, CM of the two NHL cell lines MAVER-1 and K422 induced phenotypical disorganization and reduced growth capacity in healthy MSCs. In contrast, CM from the MEC-1 NHL cell line and the NALM-6 ALL cell line did not induce these alterations in healthy MSCs (Figure 4A,B). Interestingly, CM derived from all cell lines induced a significantly reduced expression of *OSTEOCALCIN* in MSCs (Figure 4C).

Conditioned media (CM) derived from the MNCs of patients with myeloid neoplasms lead to significantly reduced growth capacity of MSCs (cell number: +CM MNC Healthy: 2.7 × 10^5^; +CM MNC Myeloid: 2.2 × 10^5^) after three days. A 2.2-fold reduced cell number of healthy MSCs after co-culture with CM derived from the MNCs from ALL and NHL patients was found and accompanied by a dramatically altered broad and flattened morphology (Figure 4D,E).

Consistent with experiments using cell line derived CM, the mRNA expression for *OSTEOCALCIN* was 22-fold significantly diminished after co-culture with CM derived from MNCs from patients with myeloid neoplasms, and 63-fold diminished after co-culture with CM derived from MNCs from patients with lymphoid neoplasms (Figure 4F).

### 3.5. RNA Sequencing Distinguish MSCs from Myeloid and Lymphoid Neoplasms

**Figure 5:**
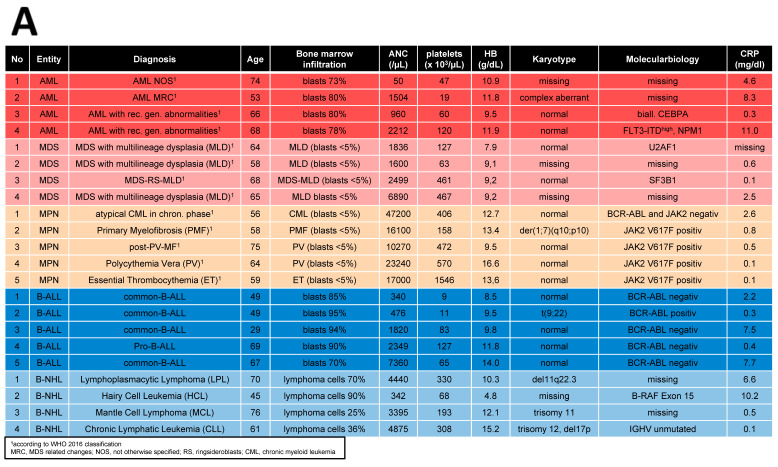

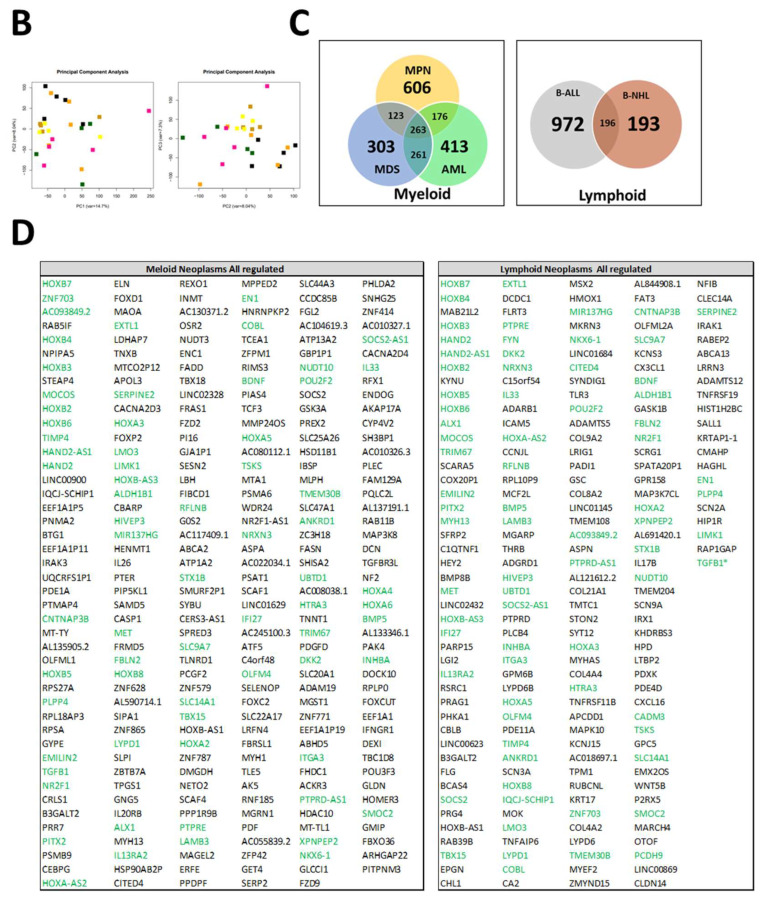
RNA Sequencing. (**A**) Clinical parameters of patients’ characteristics, which were included in RNA sequencing. (**B**) Principal Component Analysis (PCA) of MSCs from Healthy (*n* = 5, black), MPN MSCs (*n* = 5, purple), MDS (*n* = 4, brown), AML (*n* = 4, yellow), B-ALL (*n* = 5, orange), and B-NHL (*n* = 4, green). (**C**,**D**) Venn diagrams of differentially expressed genes (FDR q-value ≤ 0.05) for MSCs from myeloid (left: MPN, MDS, and AML) and for MSCs from lymphoid (right: B-ALL and B-NHL) neoplasms with tables of overlapping differentially expressed genes of 263 in MSCs of myeloid neoplasms (MPN, MDS, and AML MSCs; left) and 196 in MSCs of lymphoid (B-ALL and B-NHL; right) neoplasms. Green highlighted genes indicate overlapping genes in all hematological neoplasms and. black highlighted genes were exclusively found in the respective group (myeloid or lymphoid) (TGFB1*, was significant in *p*-value 0.005, FDR q-value 0.1, B-ALL sample 2 exhibited a lower Transcript per million (TPM) expression of TGFB1 than all other samples).

In order to obtain a better understanding of the observed functional and morphological alterations in MSCs across all entities at the molecular level and to identify common mechanisms, we performed RNA sequencing. As the respective entities are heterogeneous, we selected some representative samples from patients with B-cell neoplasms. In the case of myeloid neoplasms in AML, we concentrated on those with blasts, whereas in patients with MDS or MPN, we concentrated on those without blast proliferation. As a result, RNA sequencing data were available for MSCs from five healthy donors, four MDS, five MPN, four AML, four B-NHL, and five B-ALL. A detailed patient characteristic of the selected samples is shown in Figure 5A. In myeloid neoplasms (MDS, MPN, and AML), RNA sequencing revealed, in comparison to normal donors, 606 exclusively differentially expressed genes in MPN MSCs, 303 in MDS, and 413 in AML (FDR: q ≤ 0.05). The greatest overlap, with 261 differential genes in MSCs, was noted between the pathophysiologically closely related myeloid stem cell diseases MDS and AML. This is reflected to some extent in the result of the Principal Component Analysis (PCA), which grouped the samples of MDS and AML closer to each other than to those of the other entities (Figure 5B). In contrast, the number of differentially expressed genes overlapping between MDS and MPN was 123, while the comparison of MPN and AML MSCs reveals a total overlap of 176 differentially expressed genes. Looking at the intersection of genes differentially expressed in all three entities, 263 overlapping differentially expressed genes were found. In lymphoid neoplasms of B-cell origin (B-ALL, B-NHL), RNA sequencing showed 972 differentially expressed genes exclusively in B-ALL derived MSCs and far fewer (193) exclusively differentially expressed in B-NHL-derived MSCs in comparison to healthy controls (FDR: q ≤ 0.05). Meanwhile, there was an overlap of 196 differentially expressed genes in the MSCs of patients from both lymphoid entities.

In the next step, we focused on differences and similarities between MSCs from myeloid and lymphoid neoplasms to unravel overlapping genes and pathways that may contribute to hematopoietic insufficiency. For that purpose, we performed a detailed examination of the differentially expressed genes (FC ≥ 1.5, FDR q ≥ 0.05) across all hematological neoplasms, encompassing 263 genes for the myeloid and 196 genes for the lymphoid entities. First, we concentrated on the subset of differentially expressed genes that we only found in the myeloid neoplasms. Among those are key players such as TGFBR3L, Integrin Binding Sialoprotein (IBSP), Frizzled-2 (FZD-2), and Frizzled-9 (FZD-9), which are essential for governing osteogenesis (Figure 5C,D). On the other hand, there were differentially expressed genes which we only overserved in patients with lymphoid neoplasms. Among them are BMP8B, WNT5B, TLR3, and TNFRSF19, which are pivotal for proliferation or apoptosis. This subset of genes also contained a substantial number of genes encoding collagens (COL9A2, COL8A1, COL21A1, COL4A4, and COL4A2), which are crucial for tissue development, osteogenesis, and cartilage formation (Figure 5C,D).

### 3.6. RNA Sequencing Revealed Overlapping Genetic Signatures Associated with Chondrogenic-Osteogenic Differentiation and Hematopoietic Supporting Capacity in MSCs from Myeloid and Lymphoid Neoplasms

**Figure 6:**
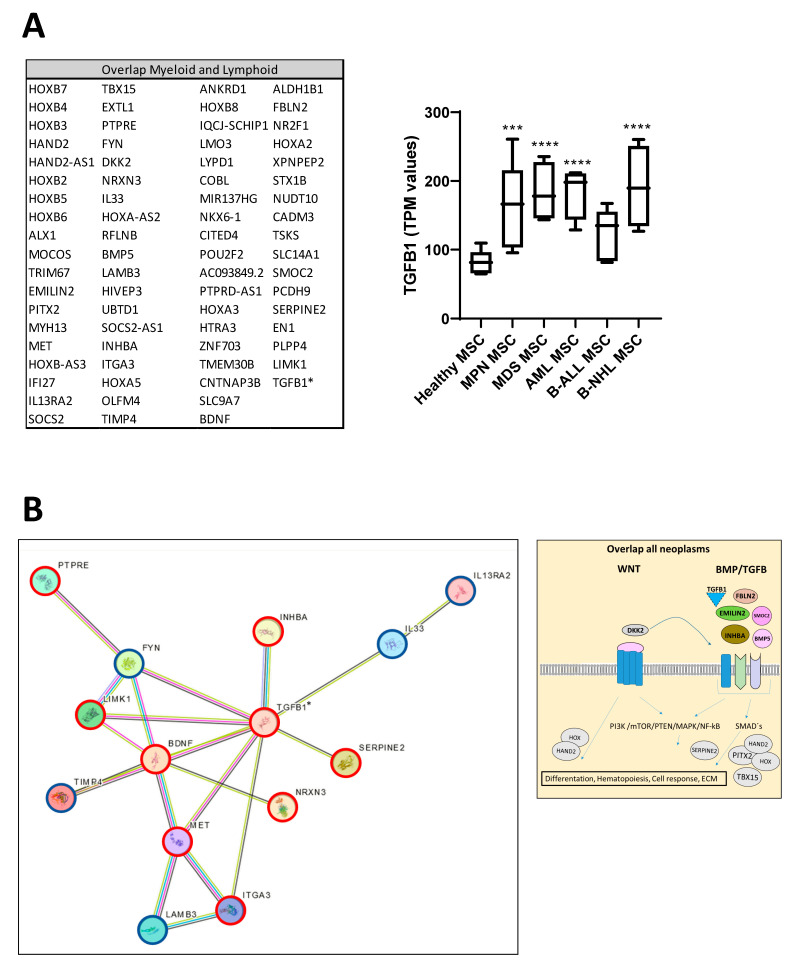

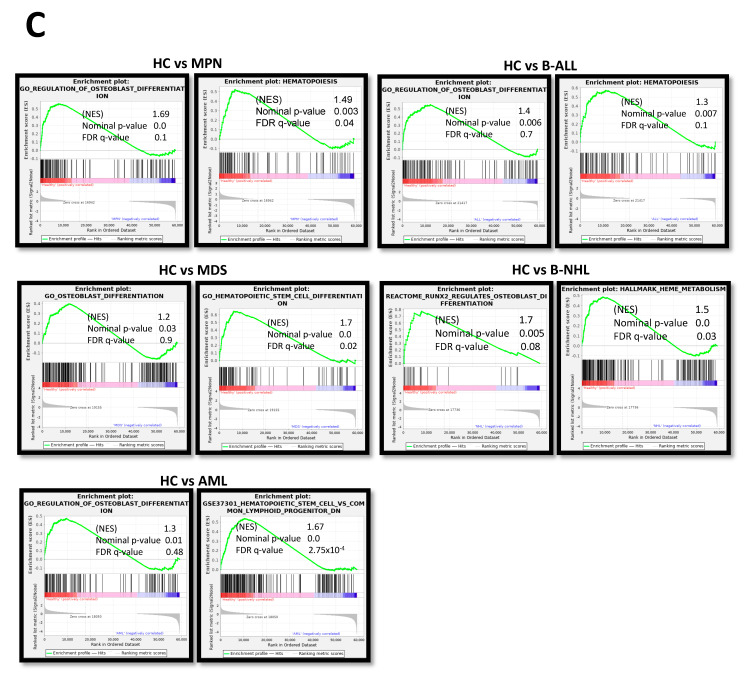
Overlapping differentially expressed genes in all hematological neoplasms affect cell processes. (**A**) Left side: Graphical overview of representative overlapping differentially expressed genes that were found in MSCs from all hematological neoplasms from overlapping genes in the respective group from green highlighted in Figure 5D. A total of 74 differentially overlapping genes were identified in all hematological neoplasms. Right side: Transcript per million (TPM) values of TGFB1 in MSCs from our RNA sequencing data. Asterisks display adjusted *p*-values *** *p* < 0.001, **** *p* < 0.0001. (**B**) A total of 14 of these 74 genes are directly linked and form a potential theoretical protein network to TGFB signaling as visualized by using the STRING database (**B**, right side). A protein network was generated with the STRING database (Version 12.0) and adapted. Blue-circled genes indicate downregulation and red-circled genes indicate upregulation of these genes in our RNA sequencing. Visualization of further genes from the “overlap all neoplasms” group, that are regulators or related to the WNT and BMP/TGFB signaling pathways as well as downstream targets of these signaling pathways such as *PITX2*, *HAND2,* or *TBX15*, that were found in all hematological neoplasms, were contrasted with healthy MSCs. These genes play crucial roles in ECM, differentiation, or hematopoiesis (TGFB1*, was significant in *p*-value 0.005, FDR q-value 0.1, B-ALL sample 2 exhibited a lower Transcript per million (TPM) expression of TGFB1 than all other samples). (**C**) Strong enrichment in genes related to osteogenesis or hematopoiesis in all MSC groups reflected by gene set enrichment analysis (GSEA). Normalized enrichment score (NES), *p*-value, and false discovery rate (FDR) are given.

Looking at the group of “overlapping genes” that were differentially expressed in all hematological neoplasms, we found candidates crucially involved in osteogenesis and hematopoiesis, such as BMP5 (Bone morphogenetic protein 5). This is a member of the TGF superfamily and a key player in osteogenesis, regulating several downstream processes including downregulated genes such as *HAND2*, the T-Box transcription factor *TBX15,* and *PITX2*, which are crucial for skeletal formation. The Dickkopf WNT signaling pathway inhibitor 2 (DKK-2) is another crucial factor for osteogenesis, with cross-talking to the BMP/TGF signaling pathway including PITX2 [21,22,23]. Furthermore, in MSCs from all hematological neoplasms, we observed an overexpression of *HOXA2* and *HOXA3* as well as *HOXB2-8* genes, which are crucial for hematopoietic cell fates and skeletal development. The group of overlapping genes also included proteins participating in the formation of extracellular matrix (ECM) such as *EMILIN2* or *TIMP4* (Metalloproteinase Inhibitor 4) (Figure 6A,B). Gene set enrichment analysis (GSEA) reveals a strong enrichment of genes for osteogenesis and hematopoiesis in MSCs derived from patients with myeloid and lymphoid neoplasms (Figure 6C). The differentially expressed genes were further corroborated through Reactome and Gene ontology (GO) analysis, encompassing processes as outlined in more detail in Supplementary Figure S1. Interestingly, looking for differences among the entities, Reactome analysis indicated a strong enrichment of genes involved in cell cycle regulation, particularly in the patients with MDS and AML. On the other hand, genes governing ECM processes were particularly affected in MSCs derived from lymphoid neoplasms. In line with this, GO analysis provides a similar tableau with a significant enrichment of genes involved in growth and cell death processes that were most affected in MDS- and AML-derived MSCs, and a strong enrichment of genes involved in skeletal system formation in MSCs from all groups (Supplementary Figure S2).

### 3.7. TGFB1 as Potential Overlapping Upstream Regulator

**Figure 7:**
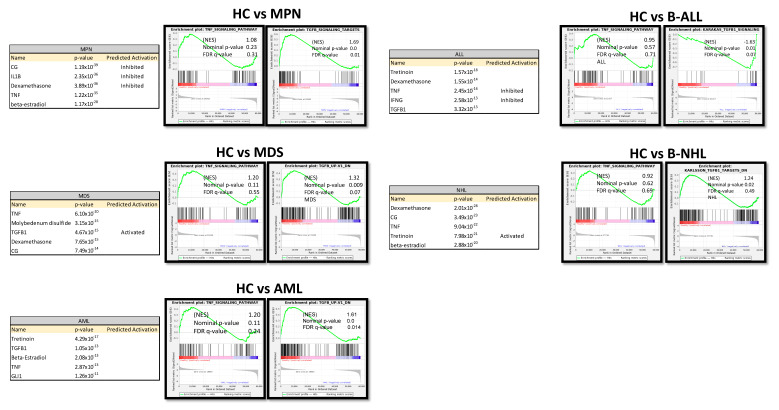
Ingenuity Pathway Analysis (IPA) and gene set enrichment analysis (GSEA) of native MSCs from Healthy, MPN, MDS, AML, ALL, and NHL MSCs. Tables of Ingenuity Pathway Analysis (IPA) and the prediction of potential upstream regulators from native MSCs from MPN, MDS, AML, B-ALL, and B-NHL contrasted to healthy and waterfall Plots from GSEA of native MSCs from Healthy, MPN, MDS, AML, B-ALL, and B-NHL MSCs. *p*-value, FDR q-value, and normalized enrichment score (NES) are included.

Ingenuity Pathway Analysis (IPA) predicted TNF or TGFB1 as potential upstream regulators in all patient-derived MSCs (Figure 7). Gene set enrichment analysis (GSEA) confirmed a strong enrichment for a TGFB signature but not for a TNFA signature in all patient-derived MSCs contrasted to healthy MSCs, except for B-NHL-derived MSCs. The latter showed a strong enrichment of an interferon signature (Figure 7).

### 3.8. RNA Sequencing Revealed SMAD-Dependent and SMAD-Independent BMP/TGFB-Signaling in Myeloid and Lymphoid Neoplasms, Which Can Both Be Blocked as Potential Therapeutic Targets

**Figure 8:**
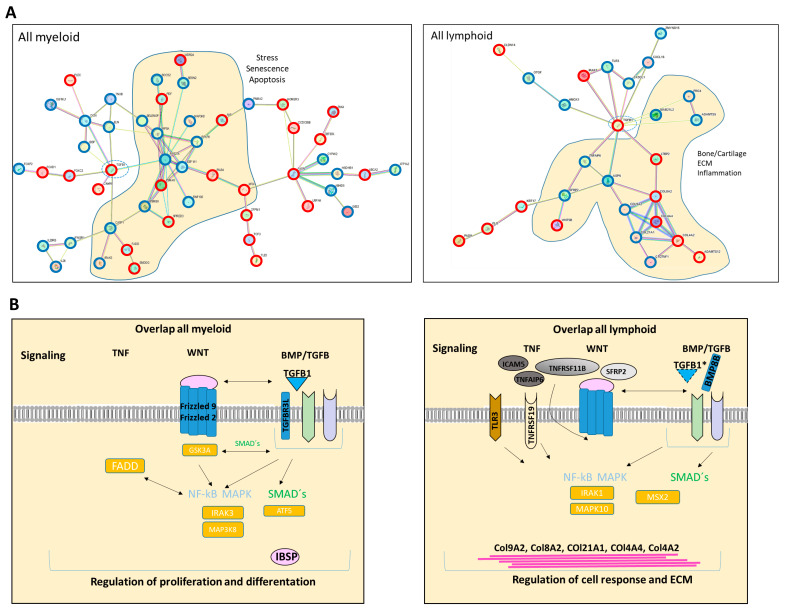

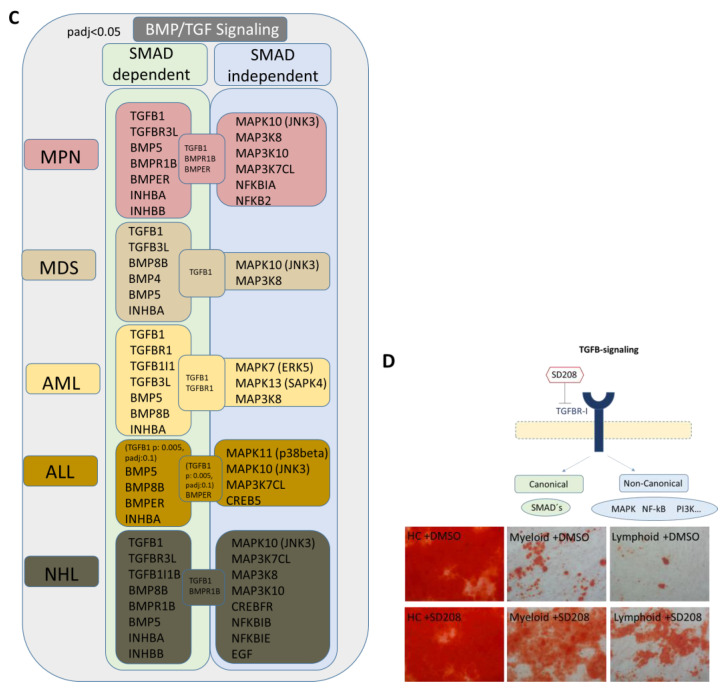
Graphical overview of representative exclusively differentially expressed genes from our RNA sequencing data in the myeloid and lymphoid group that are clearly assigned to the BMP/TGFB signaling pathway. (**A**) Illustration of representative and exclusively differentially expressed genes only in the “all myeloid group” (MPN, MDS, AML) and the exclusively overlapping differentially expressed genes only in the “all lymphoid group” (B-ALL, B-NHL) (from Figure 5D, black highlighted genes). Translation of differentially expressed genes from our mRNA sequencing data (FDR q-value < 0.05) from overlapping genes in all myeloid and separately in all lymphoid groups to a correlation and potential protein network of these genes as generated with STRING database (Version 12.0). STRING visualization was adapted starting from TGFB1 as is highlighted with a circle. In the “all myeloid group”, 52 out of 192 exclusively differentially expressed genes in this group clearly show a direct correlation to the TGFB-signaling cascade based on current data in STRING (Version 12.0) (**B**, left side). In the “all lymphoid group”, 26 out of 123 exclusively differentially expressed genes in this group clearly show a direct correlation to the TGFB-signaling cascade based on current data in STRING (Version 12.0) (**B**, right side). Overrepresented genes are red-circled, and underrepresented genes are blue-circled. (**B**) Graphical overview of further exclusively differentially expressed genes in the respective group, myeloid or lymphoid group (contrasted to healthy) that are regulators or related to the WNT and BMP/TGFB signaling pathways as well as downstream targets of these signaling pathways exclusively in the respective group. (TGFB1*, was significant in *p*-value 0.005, FDR q-value 0.1, B-ALL sample 2 exhibited a lower Transcript per million (TPM) expression of TGFB1 than all other samples). (**C**) Illustration of representative and exclusively overlapping differentially expressed genes in the “all myeloid group” and the “all lymphoid group” and the relationship to the canonical and non-canonical BMP/TGFB signaling pathway. Illustration of DESeq2 Data (*p*-adjusted < 0.05) reveals a clear shift to SMAD-dependent BMP/TGFB signaling in MDS and AML MSCs, while B-ALL, B-NHL, and the MPN group exhibit an increase and notable number of differentially expressed genes related to SMAD independent MAPK-signaling. (**D**) Representative pictures of MSCs from healthy, myeloid, and lymphoid groups after 14 days of osteogenic differentiation together with SD208. DMSO serves as an internal control. MSCs were induced for osteogenic differentiation and SD208 was added to each medium change within the osteogenic differentiation period. After 14 days of induction, MSCs were stained with Alizarin Red. Scale bars indicating 100 µm are shown.

We were able to detect overlapping differentially expressed genes in the myeloid and lymphoid groups that are related to growth, differentiation, and hematopoiesis. BMPs, INHBA, or DKK2 could be clearly assigned to the WNT and BMP/TGFB signaling pathways. Exclusively differentially expressed genes related to the BMP/TGFB signaling pathway in the respective group indicated a shift in the regulated signaling pathways. In the next step, we were interested in other genes related to these signaling pathways and whether we could find a group-specific signature. In the myeloid samples, a considerable number of genes integrated into or regulated by the canonical BMP/TGFB and WNT signaling pathways, as well as TGFB1, Frizzled, or GSK3A, were directly cross-talking and regulating downstream targets by SMAD- and beta-catenin signaling (Figure 8A,B). In comparison, the lymphoid group showed an impressive number of specifically differentially expressed collagens regulated by inflammatory programs, such as the MAPK pathway induced by TGFB (Figure 8A,B). A detailed view into our DESeq2 Data revealed a clear shift to canonical BMP/TGFB-signaling in MSCs from myeloid neoplasms, especially for MDS and AML, reflected by a respective number of genes such as TGFB1, TGFB3L, and a low number of detected genes for MAPK-signaling (via JNK and ERK). The heterogeneous MPN group exhibited a high number of both genes involved in SMAD-dependent and SMAD-independent BMP/TGFB signaling. A clear increase in differentially expressed genes involved in TGFB-inducible MAPK-signaling was detected in ALL and a higher number in NHL MSCs, as reflected by MAPK11 or MAPK10 (Figure 8C). Assuming that TGF/BMP-signaling acts as an overarching regulator of the canonical and non-canonical signaling pathway in varying degrees in the individual entities and is deregulated here, we wondered whether a blockade could reverse the observed effects. Since we saw overlapping impairments in the MSCs of various entities, particularly in osteogenic differentiation, we induced osteogenic differentiation in healthy MSCs and in MSCs derived from myeloid and lymphoid neoplasms, and added the active ATP-competitive transforming growth factor-β receptor 1 (TGF-βRI) (ALK5) inhibitor SD208 to each medium change within the osteogenic differentiation period. Interestingly, the addition of SD208 during the osteogenic differentiation period led to a significant restoration of osteogenic differentiation capacity (Figure 8D).

## 4. Discussion

The major finding of this comprehensive study on the alterations inferred from the mesenchymal stromal cells of the bone marrow by infiltrating malignant cells of hematological neoplasms is diverse and dependent on the type of neoplasm. This is true with regard to the phenotypical changes as well as the molecular signature as reflected by the results of RNA sequencing of MSCs from myeloid and lymphoid neoplasms.

For instance, in MSCs derived from the myeloid neoplasms, a remarkable number of genes belonging to the TGF superfamily genes, such as TGFB-1, various BMPs, and INHBA as well as genes of the WNT signaling pathway, were differentially expressed. MPN-derived MSCs were the most heterogeneous group, showing a high number of additional differentially expressed genes related to inflammation or extracellular matrix (ECM) formation. In particular, the genes from the BMP/TGF signaling pathway are crucial for the regulation of proliferation, cell cycle, or differentiation of bone marrow MSCs. This finding may explain the profound functional deficits—significantly greater in comparison to the MSCs derived from the lymphoid group—such as structural integrity, cellular senescence, osteogenic differentiation capacity, and long-term culture initiating cell capacity. The latter deficit is characterized by a lack of *SDF-1* and *CXCL12* expression. These stroma-derived growth factors are essential for the maintenance of normal hematopoietic stem cells within their bone marrow niche. In accordance with these findings, it was shown by another group in myeloproliferative neoplasms that the hematopoietic cells induced a downregulation of the HSPC retention marker *CXCL12* [12]. The architecture of this particular niche also greatly depends on osteoblast and on a finely tuned osteogenic differentiation of MSCs which is significantly hampered in the myeloid-derived MSCs. These findings provide a reasonable explanation of the hematopoietic insufficiency observed with the strongest impairment encountered in patients with MDS and AML. On the other hand, in MSCs derived from lymphoid neoplasms, a remarkable number of genes involved in inflammatory processes like the TNF signaling pathway or the NFKB signaling pathway were observed. Different from the myeloid group, we found a high number of differentially expressed genes encoding proteins involved in the formation of the extracellular matrix (ECM), in particular members of the collagen family. Some studies have shown that MSCs from B-ALL patients have a pro-inflammatory genetic signature. The authors also found that MSCs from these patients were more prone to senescence and had a reduced osteogenic differentiation capacity, while their adipogenic and chondrogenic differentiation capacity was normal or increased [24,25,26]. In comparison, we found that the molecular disturbance with regard to growth and differentiation along the osteogenic and chondrogenic lineage was similar to ALL patients. In this context, other studies such as Medina et al. interestingly showed that MSCs from patients with mantle cell lymphoma protect the malignant cells from spontaneous and drug-induced apoptosis through the secretion of B-cell activating factor and activation of the canonical and non-canonical NFκB pathways [27]. Approaching the group of “overlap genes” which were in comparison to the healthy controls differentially expressed in all hematological neoplasms, we found candidates, such as *DKK2*, *PITX2*, and *TBX15*, various *HOXA2/3, HOXB2-B8* genes, and *BMP5,* that are crucially involved in the regulation of cell growth, osteogenesis, and hematopoiesis. For instance, BMP5 as a member of the TGF superfamily plays a pivotal role in the regulation of various processes, and is crucial for the stimulation of chondrogenesis and osteogenesis. Downstream targets such as *PITX2* or osteogenic transcription factors such as *OSTERIX* play an essential role in osteogenic differentiation. In addition, BMPs are known to regulate the expression of other factors such as *SERPINE2* involved in ECM formation, while, for example, HOXA2 inhibits *BMP5* expression in murine palatal MSCs. Altogether, alterations in gene expression and signaling pathways reflect a dysregulated osteogenesis contributing to a dysfunctional stem cell niche, irrespective of the underlying hematological disease [23,28,29].

Again, these molecular data are in accordance with the results of our in vitro cultures, demonstrating that MSCs derived from all hematologic neoplasms exhibit reduced osteogenic differentiation capacity, in particular going along with a reduced mRNA expression of the osteogenic marker *OSTEOCALCIN*. Assuming soluble factors have the potential to induce the observed functional alterations, we could show that exposure of healthy MSCs to patient-derived conditioned media led to a phenotypical disorganization and reduced growth capacity, and induced a reduced expression of *OSTEOCALCIN* in these MSCs. What are the mechanisms by which the BM-infiltrating cells confer their malignant imprint on the normal stroma cells? Based on the mRNA sequencing and functional data, we consider genes of the TGF family as dominant players in the pathophysiological network for hematopoietic insufficiency in all hematological neoplasm. In that respect, the findings of our comprehensive study confirm our previous findings of mRNA sequencing data from the MSCs of patients with MDS, AML, or multiple myeloma, and are in line with the results from other groups. These published data show that the structural and functional deficits greatly result from the secretion of soluble factors TGFB or CCL-3. They exert direct inhibitory and indirect effects resulting in the molecular reprogramming of the MSCs in myeloid and lymphoid neoplasms [9,10,13,14,15,16,17,18,30,31,32]. Accordingly, upon exposure to TGFB1, healthy MSCs developed functional deficits and adopted a phenotype reminiscent of that observed in patient-derived stromal cells, which could be reverted by the TGFB1 inhibitor SD208, especially towards their osteogenic differentiation capacity (Figure 4C,E) [9,16]. Despite the involvement of genes and pathways common for both groups of hematological malignancies, it appears that disturbances along the canonical signaling pathway are predominant in myeloid neoplasms, whereas alterations along the non-canonical signaling pathway are more prominent in lymphoid neoplasms as reviewed by other groups [33,34,35,36]. It was therefore not surprising that blocking TGFB signaling with SD208, which inhibits both the canonical and non-canonical pathways, led to an improvement in functionality, especially osteogenic differentiation in both groups.

## 5. Conclusions

In summary, mesenchymal stromal cells (MSCs) derived from patients with myeloid neoplasms (MPN, MDS, and AML) as well as lymphoid neoplasms (B-ALL and B-NHL) exhibit structural and functional alterations. Consequently, they play a crucial role in the pathophysiology of these conditions, significantly contributing to inadequate hematopoiesis. Our findings suggest the presence of overlapping mechanisms not only in the phenotype and functionality of MSCs but also in the underlying molecular pathways. Commonly involved factors, such as members of the TGF superfamily, have been identified across all neoplasms. Therefore, effectively blocking these pathways represents a promising strategy for improving hematopoiesis in affected patients.

## Figures and Tables

**Table 1:** Patient demographics and clinical characteristics.

	No.	%
Patients No.	70	
**Sex**		
Male	42	60
Female	28	40
**AML No.**	**25**	
Median age, years (Range)	61	(25–74)
Diagnosis WHO 2016 ^1^		
Median BM infiltration, % (Range)	69	(35–95)
Median ANC, ×10^3^/µL (Range)	16.50	(0.00–21.00)
Median platelets, ×10^3^/µL (Range)	60	(6–279)
Median HB, g/dL (Range)	9.4	(4.2–11.9)
**MDS No.**	**16**	
Median age, years (Range)	68	(47–81)
Diagnosis WHO 2016 ^2^		
Median BM infiltration, % (Range)	Dysplasia ^2^, blasts > 5% in 6	
Median ANC, ×10^3^/µL (Range)	1.76	(0.20–6.89)
Median platelets, ×10^3^/µL (Range)	69	(27–467)
Median HB, g/dL (Range)	9.2	6.6–11.2
**MPN No.**	**11**	
Median age, years (Range)	60	(22–75)
Diagnosis WHO 2016 ^3^		
Median BM infiltration, % (Range)	Proliferation, blasts < 5%	
Median ANC, ×10^3^/µL (Range)	16.10	(5.40–47.20)
Median platelets, ×10^3^/µL (Range)	534	(54–1564)
Median HB, g/dL (Range)	13.6	(9.3–16.6)
**ALL No.**	**9**	
Median age, years (Range)	47	(22–69)
Diagnosis ^4^		
Median BM infiltration, % (Range)	85	(40–95)
Median ANC, ×10^3^/µL (Range)	2.35	(0.34–15.20)
Median platelets, ×10^3^/µL (Range)	53	(9–138)
Median HB, g/dL (Range)	11.8	(8.5–16.7)
**NHL No.**	**9**	
Median age, years (Range)	63	(38–76)
Diagnosis ^5^		
Median BM infiltration, % (Range)	25	(10–90)
Median ANC, ×10^3^/µL (Range)	3.40	(0.33–8.90)
Median platelets, ×10^3^/µL (Range)	193	(68–345)
Median HB, g/dL (Range)	12.5	(4.8–15.2)

^1^ AML with rec. gen. abnormalities (*n* = 11), AML MRC (*n* = 7), AML NOS (*n* = 7). ^2^ MDS-MLD (*n* = 7), MDS-EB-I (*n* = 4), MDS-EB-II (*n* = 2), MDS-RS-MLD (*n* = 3). ^3^ PV (*n* = 2), ET (*n* = 2), PMF (*n* = 2), atyp. CML (*n* = 1), CML (*n* = 1), MPN-U (*n* = 1), post-PV-MF (*n* = 1), post-ET-MF (*n* = 1). ^4^ precursor-B-ALL (*n* = 6), precursor-T-ALL (*n* = 1), mature-T-ALL (*n* = 2). ^5^ LPL (*n* = 3), MCL (*n* = 2), HCL (*n* = 2), B-CLL (*n* = 1), MZL (*n* = 1). Abbreviations: AML, Acute Myeloid Leukemia; ANC, Absolute Neutrophil Count; ALL, Acute Lymphoblastic Leukemia; BM, Bone Marrow; CLL, Chronic Lymphatic Leukemia; CML, Chronic Myeloid Leukemia; EB-I, with Excess Blasts 5–9% in BM; EB-II, with Excess Blasts 10–19% in BM; ET, Essential Thrombocythemia; HB, Hemoglobin; HCL, Hairy Cell Leukemia; LPL, Lymphoplasmacytic Lymphoma; MCL, Mantle Cell Lymphoma; MF, Myelofibrosis; MDS, Myelodysplastic Syndrome; MLD, Multilineage Dysplasia; MRC, MDS Related Changes; MPN, Myeloproliferative Neoplasia; MZL, Marginal Zone Lymphoma; NOS, Not Otherwise Specified; PMF, Primary Myelofibrosis; PV, Polycythemia Vera; RS, Ringsideroblasts; U, Unclassified.

## Data Availability

RNA-seq expression data are currently being deposited in the NCBI Sequence Read Archive (SRA) with BioProject ID PRJNA1091937.

## Supplementary Materials

The following supporting information can be downloaded at: https://www.mdpi.com/article/10.3390/cancers16112071/s1, Figure S1: Reactome analysis of MSCs derived from MPN, MDS, AML, ALL, and NHL; Figure S2: Gene ontology (GO) analysis of MSCs derived from myeloid and lymphoid neoplasms contrasted to healthy MSCs.
