# Supplementary material for: Overlapping Stromal Alterations in Myeloid and Lymphoid Neoplasms
Source: Cancers (Basel). 2024 May 30;16(11):2071. doi: 10.3390/cancers16112071 (PMC11171322; doi:10.3390/cancers16112071)
Supplement: Supplementary file 1 [file cancers-16-02071-s001.zip › cancers-2996780-supplementary.pdf]

cancers

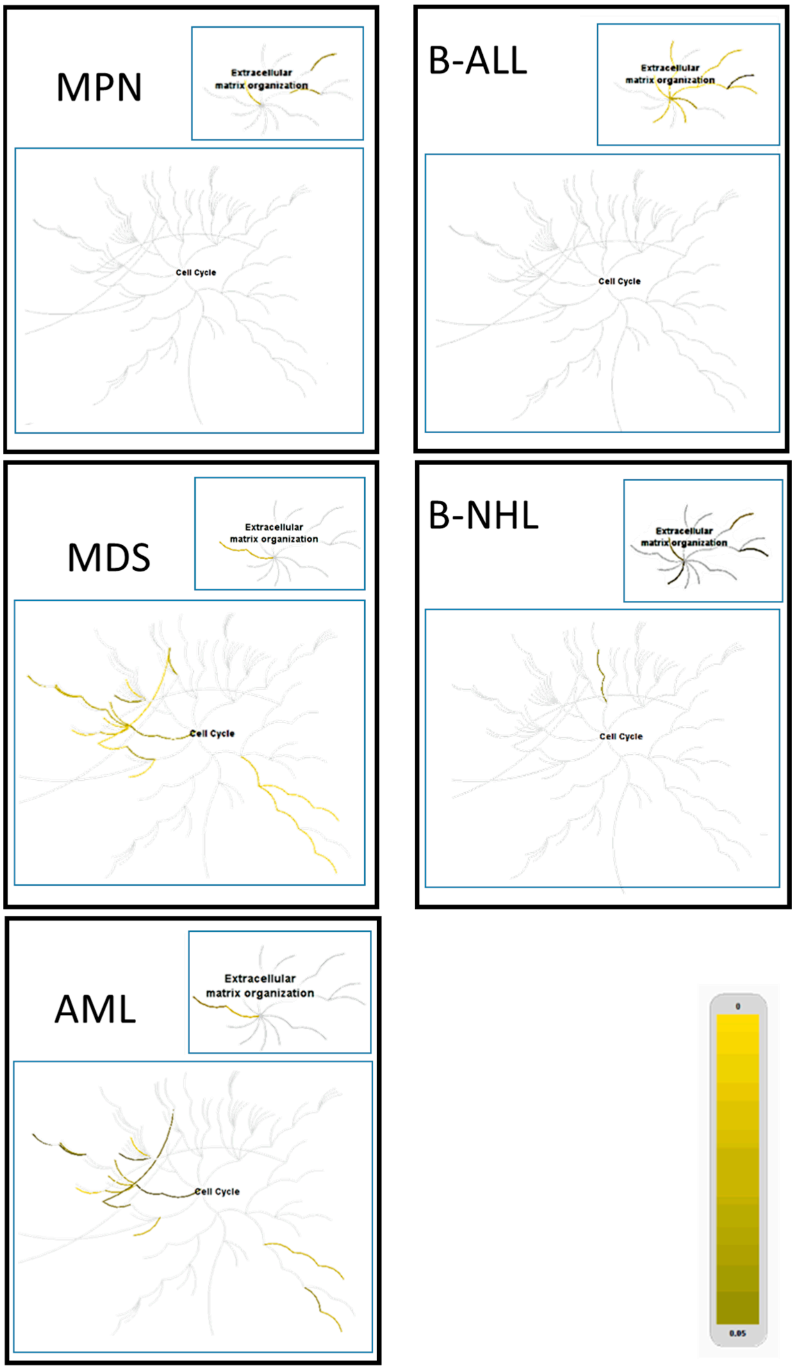

Supplementary Figure S1. Reactome analysis of MSCs derived from MPN, MDS, AML, ALL, and NHL. A strong enrichment of cell cycle was detected in MDS- and AML-derived MSCs from the myeloid group, while MPN, B-ALL, and B-NHL-derived MSCs exhibited a strong enrichment for extracellular matrix (ECM).

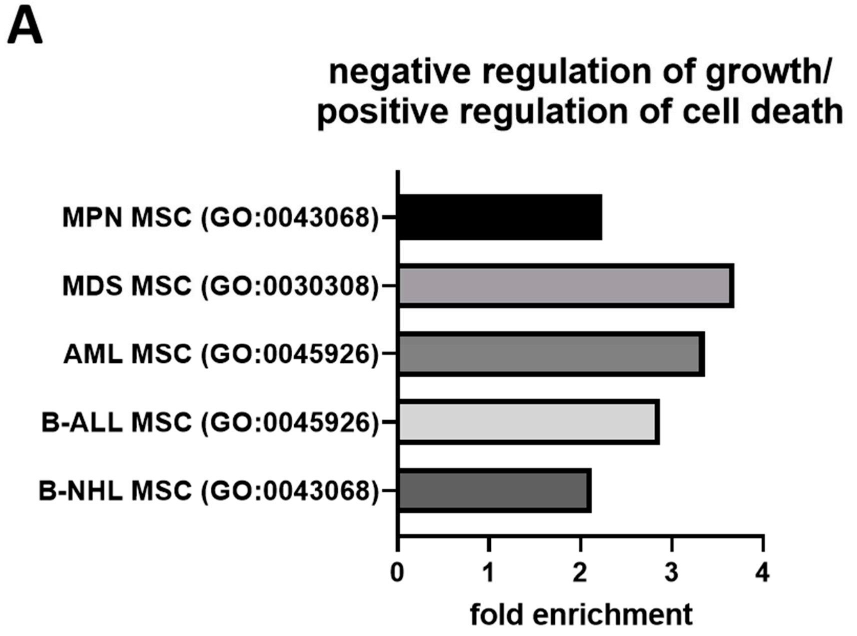

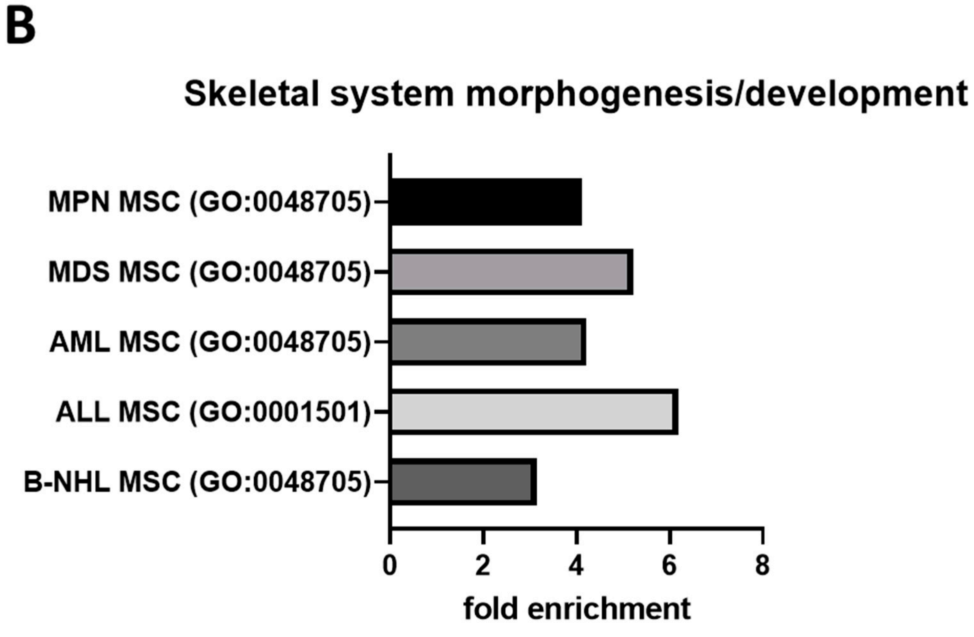

Supplementary Figure S2. Gene ontology (GO) analysis of MSCs derived from myeloid and lymphoid neoplasms contrasted to healthy MSCs. (A) Representative GO analysis for enriched cell processes such as overrepresented negative regulation of growth/positive regulation of cell death (GO:0043068 regulation of cell death; GO:0030308 negative regulation of cell growth; GO0045926 negative regulation of growth) were strongly enriched in all patient groups, with the highest fold-change in MDS and AML MSCs. (B) Representative GO analysis for underrepresented skeletal system morphogenesis/development (GO:0048705 skeletal system morphogenesis; GO:0001501 skeletal system development). MSCs from both myeloid and lymphoid patient groups exhibit a significant strong enrichment in the skeletal system.
